# T160. TREATMENT OF CLOZAPINE-ASSOCIATED OBESITY AND DIABETES WITH EXENATIDE (CODEX) IN ADULTS WITH SCHIZOPHRENIA: A RANDOMISED CONTROLLED TRIAL

**DOI:** 10.1093/schbul/sby016.436

**Published:** 2018-04-01

**Authors:** Dan Siskind, Anthony Russell, Clare Gamble, Karl Winckel, Sam Hollingworth, Steve Kisely

**Affiliations:** 1 Metro South Addiction and Mental Health Service; 2 University of Queensland; 3 University of Queensland, MSAMHS

## Abstract

**Background:**

Clozapine is the most effective anti-psychotic for treatment refractory schizophrenia, but causes significant metabolic disturbances including obesity and type 2 diabetes. The metabolic adverse reactions may be mediated in part by clozapine induced dysregulation of Glucagon-like-peptide-1 (GLP-1). GLP-1 is an intestinal epithelial derived peptide, released with ingestion of food, that triggers satiety, reduces glucagon production, promotes insulin production and slows gut motility. Clozapine has been shown to interfere with GLP-1 function in animal models, leading to metabolic dyregulation including obesity and preference for high calorie meals. Administration of exogenous GLP-1 agonists such as exenatide to animals have been shown to counter this effect of clozapine. Exenatide subcutaneous weekly injections may assist obese people on clozapine lose weight.

**Methods:**

This randomised, controlled, open-label, pilot trial aimed to evaluate the effect of exenatide on weight loss among clozapine-treated obese adults who have schizophrenia, with or without stable diabetes. Twenty-eight out-patients were randomised to once weekly extended release sub-cutaneous exenatide or treatment as usual for 24 weeks. This trial examined the safety, tolerability and acceptability of exenatide among obese people with schizophrenia on clozapine, with an evaluation of change in weight, glycaemic control, psychosis severity and metabolic parameters.

**Results:**

All 28 participants completed the study. (Exenatide=14 (3 T2DM), control=14 (2 T2DM)). Six people on exenatide achieved >5% weight loss, compared to only 1 control (p=0.029). Mean weight loss was greater for exenatide than control at week 24 (-5.29kg vs -1.12kg, p=0.015) as were. BMI (-1.78 vs -0.39 p=0.019), fasting glucose (-0.34 vs 0.39, p=0.036) and HbA1c (-0.21 vs 0.03, p=0.004). There was no significant difference for other metabolic syndrome components. There were higher rates of transient nausea (n=8), vomiting (n=7) and diarrhoea (n=7) in the exenatide group.

**Discussion:**

Exenatide is a promising therapeutic agent for glycaemic control and weight loss in clozapine-treated people with obesity. These results suggest good tolerability and a consistent and favourable pattern of weight loss effects with exenatide. GLP-1 agonists could assist in reducing the cardio-metabolic associated morbidity and mortality secondary to clozapine.

